# Complete Chloroplast Genome of *Nicotiana otophora* and its Comparison with Related Species

**DOI:** 10.3389/fpls.2016.00843

**Published:** 2016-06-14

**Authors:** Sajjad Asaf, Abdul L. Khan, Abdur R. Khan, Muhammad Waqas, Sang-Mo Kang, Muhammad A. Khan, Seok-Min Lee, In-Jung Lee

**Affiliations:** ^1^School of Applied Biosciences, Kyungpook National UniversityDaegu, South Korea; ^2^Chair of Oman's Medicinal Plants and Marine Natural Products, University of NizwaNizwa, Oman; ^3^Department of Agriculture, Abdul Wali Khan University MardanMardan, Pakistan

**Keywords:** *Nicotiana*, cp genome, repeat analysis, phylogeny, sequence divergence, SSRs

## Abstract

*Nicotiana otophora* is a wild parental species of *Nicotiana tabacum*, an interspecific hybrid of *Nicotiana tomentosiformis* and *Nicotiana sylvestris*. However, *N. otophora* is least understood as an alternative paternal donor. Here, we compared the fully assembled chloroplast (cp) genome of *N. otophora* and with those of closely related species. The analysis showed a cp genome size of 156,073 bp and exhibited a typical quadripartite structure, which contains a pair of inverted repeats separated by small and large single copies, containing 163 representative genes, with 165 microsatellites distributed unevenly throughout the genome. Comparative analysis of a gene with known function across *Nicotiana* species revealed 76 protein-coding sequences, 20 tRNA sequences, and 3 rRNA sequence shared between the cp genomes. The analysis revealed that *N*. *otophora* is a sister species to *N*. *tomentosiformis* within the *Nicotiana* genus, and *Atropha belladonna* and *Datura stramonium* are their closest relatives. These findings provide a valuable analysis of the complete *N*. *otophora* cp genome, which can identify species, elucidate taxonomy, and reconstruct the phylogeny of genus *Nicotiana*.

## Introduction

Chloroplasts contain a circular DNA with approximately 130 genes, with a size ranging from 72 to 217 kb (Sugiura, 1995; Moore et al., 2007). Most cp genomes have a typical quadripartite structure consisting of a small single copy region (SSC), large single copy region (LSC), and a pair of inverted repeats (IRs) (Yurina and Odintsova, 1998; Wang et al., 2015). These inverted repeats (IRs) might influence the length of various cp genomes (Chang et al., 2006; Guisinger et al., 2011). The chloroplast (cp) DNA of green plants is exceptionally conserved in gene content and organization, providing sufficient resources for genome-wide evolutionary studies. Recent efforts have demonstrated the potential to resolve phylogenetic relationships at different taxonomic levels, and understand structural and functional evolution, by using the whole chloroplast genome sequences (Jansen et al., 2007; Moore et al., 2010). Because of the generally conservative nature of the cp genome structure, cp genome data is used most often to address phylogenetic and evolutionary questions at or above the species level.

Tobacco leaf is one of the most economically important parts of the common tobacco plant (Occhialini et al., 2016). Analyzing the composition and structure of the cp genome for such an economically important crop can explore novel genetic and evolutionary variations, which could improve plant traits (Jin and Daniell, 2015). Of the tobacco species, *Nicotiana tabacum* is one of the most widely grown commercial crops in different regions of the world. It is also a typical model organism for research in basic and important biological processes (Zhang et al., 2011). *Nicotiana tabacum* provides a key source of BY-2 plant cell lines for molecular research studies related to plant pathology and disease resistance (Nagata et al., 1992). Furthermore, considerable interest has focused on understanding the origin, organization and evolution of *N*. *tabacum* genome. It is stands out as a complex allotetraploid with large genome 4.5 GB with significant proportion of repeats (Renny-Byfield et al., 2011). As a species, *N. tobacum* evolved through the interspecific hybridization of the ancestors of *Nicotiana sylvestris* (maternal donor and *Nicotiana tomentosiformis* (paternal donor) about 200,000 year ago (Leitch et al., 2008). However, based on mitochondria and chloroplast sequence data, the chromosome segregation morphology of the flowers, and the presence of an S genome in tobacco, is thought to originate from the *N*. *sylvestris* ancestor (Sperisen et al., 1991; Murad et al., 2002). Development in modern genomics and the genome sequences of modern varieties of ancestral species were previously reported (Sierro et al., 2013), and limited evidence suggests that *N. otophora* is an alternative paternal donor (Gazdova et al., 1995; Riechers and Timko, 1999).

Plastids of *N. otophora* leaf tissue are fundamental organelles for photosynthesis and metabolic functioning. These are thought to have originated through endosymbiosis of free-living cyanobacteria with eukaryotic cells (Rodriguez-Ezpeleta et al., 2005), and remnants of cyanobacterial genes were transferred to the nucleus (Timmis et al., 2004). The angiosperm plastome has a uniparental inheritance and stable structure, making it a more informative and valuable source for phylogenetic analysis at different taxonomic levels (Ravi et al., 2008) than are mitochondrial genomes (Timmis et al., 2004). Previously, phylogenetic analyses were based on sequencing one or a few loci from plastomes of various taxa. The availability of complete chloroplast sequences, and advances in next generation sequencing techniques, has made whole plastome analysis achievable with greater and more valuable information, which could produce noteworthy results, and reduce sampling error (Martin et al., 2005). This whole genome approach may help clarify previous ambiguous phylogenetic relationships (Jansen et al., 2007; Moore et al., 2010). Recently, high–throughput sequencing technologies enabled the sequencing of hundreds of plastid genomes for terrestrial plants (Wu, 2015). Therefore, various organelle genomes from various important medicinal plants have been reported, and some are still being analyzed (Michael and Jackson, 2013).

In this study, we sequenced and analyzed the first complete chloroplast genome of *N*. *otophora*. The complete cp genome of *N. otophora*, in conjunction with previously reported cp genomes sequences, will improve our understanding of the evolutionary history of *Nicotiana* genus within *Solanaceae*, especially regarding the position of *N*. *otophora* in evolution and plant systematics. Hence, we analyzed the fully assembled chloroplast (cp) genome of *N. otophora* and compared its relationship with closely related species, such as *N. tomentosiformis, N. tabacum, N. sylvestris*, and *N. undulata*.

## Materials and methods

### Genome sequencing and assembly

A standard protocol of DNA extraction was followed as described in detailed by Sierro et al. (2014). The pure DNA was sequenced using on an Illumina HiSeq-2000. About 67,460,219 raw reads were demultiplexed, trimmed and filtered using CLC Genomics Workbench v7.0 (CLC Bio, Aarhus, Denmark). Filtered reads were assembled using *N*. *tabacum* (NC001879) as a reference genome by following the method described by Wu (2015, 2016).

### Genome annotation and sequence statistics

The online program (DOGMA) was used to annotate the *N*. *otophora* cp genome (Wyman et al., 2004). The annotation results were checked manually and codon positions were adjusted by comparing to a previously homologs gene from various chloroplast genomes present in the database. Furthermore, the tRNAscan-SE version1.21 (Schattner et al., 2005) was used to verify all transfer RNA genes using default settings. The OGDRAW program (Lohse et al., 2007) was used to draw a circular map of the *N. otophora* cp genome. GC content and codon usage were analyzed by the MEGA 6 software (Kumar et al., 2008). The mVISTA software was used to compere the *N*. *otophora* cp genome with four other cp genomes using the *N. otophora* annotation as reference (Frazer et al., 2004).

### Repeat sequence characterization and SSRs

To identify repeat sequences, including palindromic, reverse, and direct repeats within the cp genome, REPuter software was used (Kurtz et al., 2001). The following conditions for repeat identification were used in REPuter: (1) Hamming distance of 3, (2) 90% or greater sequence identity, (3) and a minimum repeat size of 30 bp. Phobos software (Leese et al., 2008) was used to detect (SSRs) within the cp genome, with the parameters set at ten repeat units ≥10 for mononucleotides, eight repeat units ≥8 for dinucleotides, four repeat units ≥4 for trinucleotides and tetranucleotides, and three repeat units ≥3 for pentanucleotide and hexanucleotide SSRs. Furthermore, tandem repeats in the *N. otophora* cp genome were identified using the Tandem Repeats Finder version 4.07 b (Benson, 1999), with default settings.

### Chloroplast genome analysis by sliding window

After aligning the sequences using MAFFT (Katoh and Standley, 2013), BioEdit software (http://www.mbio.ncsu.edu/bioedit/bioedit.html) was used to adjust the sequences manually. Furthermore, a sliding window analysis was conducted for variability (Pi) evaluation in LSC, SSC, and IR regions of the cp genome using the DnaSP version 5.1 software (Librado and Rozas, 2009). The step size was set to 200 bp, with a 600-bp window length.

### Sequence divergence and phylogenetic analysis

We used LSC, SSC, and IR regions to analyze the average pair wise sequence divergence for four *Nicotiana* species: *N*. *sylvestris, N*. *tabacum, N*. *tomentosiformis*, and *N*. *undulata* cp genomes. The missing and ambiguous gene annotations were reconfirmed by comparative sequence analysis after a multiple sequence alignment and gene order comparison. These regions were aligned using the Clustal W software (Thompson et al., 1994). Furthermore, Kimura's two parameter (K2P) model was selected to calculate the pairwise sequence divergences (Kimura, 1980). To elucidate the *N*. *otophora* phylogenetic position within the *Solanaceae* family, multiple alignments were performed using 75 protein-coding genes shared by the cp genomes of 12 *Solanaceae* members representing five genera. Two species, *Citrus aurantifolia* and *Citrus sinensis*, were designated as out-groups. Maximum parsimony (MP) analysis was executed using MEGA 6 (Tamura et al., 2013), and for Maximum likelihood (ML) analysis, the GTR + I + G nucleotide substitution model was selected. Furthermore, Bayesian inference (BI) was implemented with MrBayes 3.12 using setting (MCMC algorithm for 1,000,000 generations with 4 incrementally heated chains, starting from random trees and sampling one out of every 100 generations) from Wu et al. (2015).

## Results and discussion

### Chloroplast genome organization of *N. otophora*

**Figure 1:**
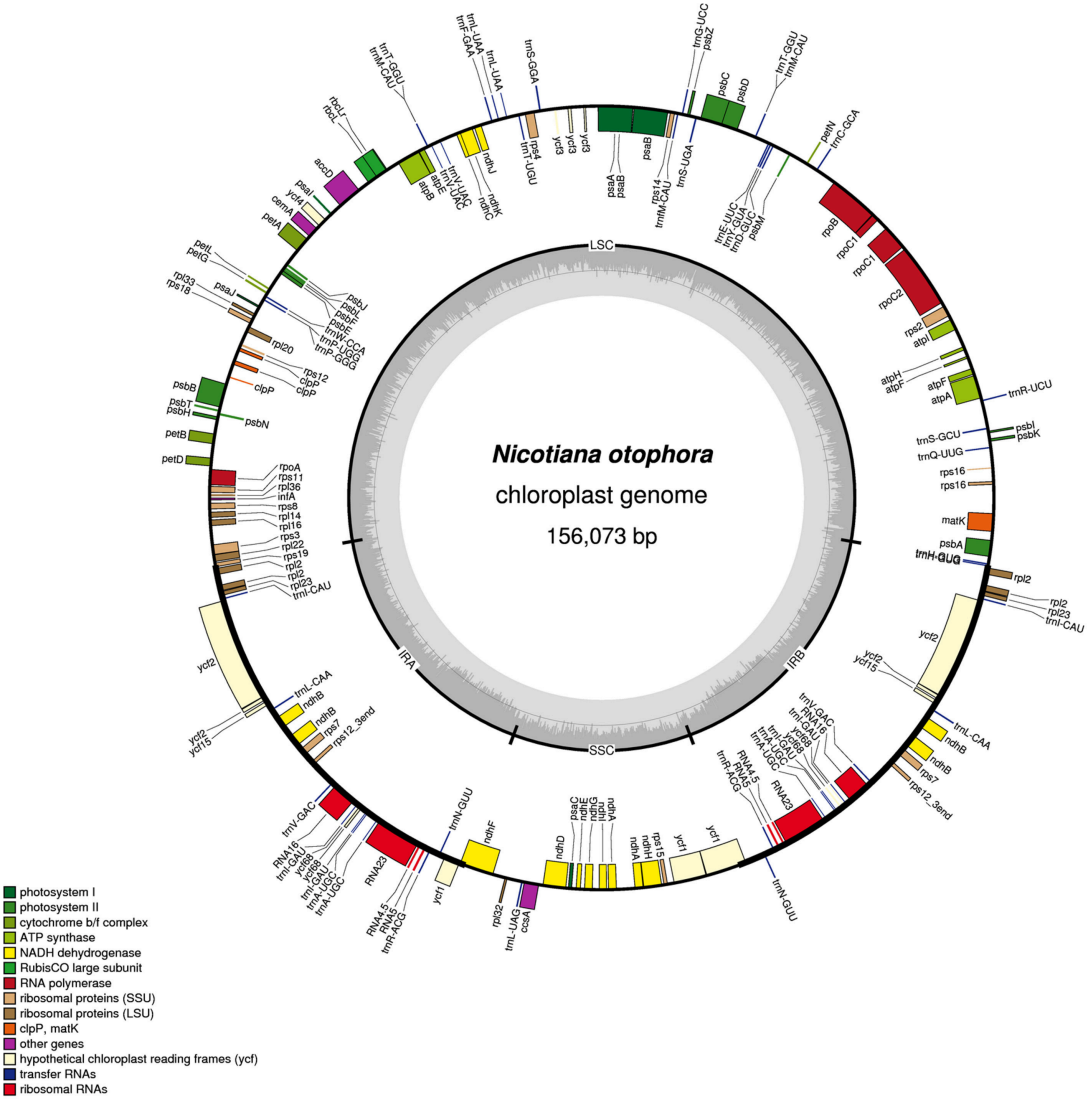
**Gene map of the *N. otophora* chloroplast genome**. Genes drawn inside the circle are transcribed clockwise, and those outside are counterclockwise. Genes belonging to different functional groups are color-coded. The darker gray in the inner circle corresponds to GC content, and the lighter gray corresponds to AT content.

*N*. *otophora* Cp genome were assembled by mapping all Illumina reads to the draft cp genome sequence, using CLC Genomics Workbench v7.0. A total of 1,877,281 reads were obtained, with an average length of 101 bp, thus yielding 341.885x coverage of the cp genome. The consensus sequence for a specific position was generated by assembling reads mapped to the position and used to construct the complete sequence of *N*. *otophora* cp genome. The size of the complete *N*. *otophora* cp genome (156,073 bp) was found to be within the range of other angiosperms (Yang et al., 2010). The cp genome exhibited a distinctive quadripartite structure, which includes a pair of inverted repeats (IRa and IRb 25,888 bp), and separate SSC (17677 bp) and LSC (86621 bp) regions (Table 1, Figure 1). The GC content (37.7%) of the *N. otophora* cp genome is very similar to other *Nicotiana* species cp genomes (Table 1; Sugiyama et al., 2005; Yukawa et al., 2006). The GC contents of the LSC and SSC regions (35.8 and 32%) are lower than that of the IR regions (43%). This high GC percentage in the IR regions is due to the presence of eight ribosomal RNA (rRNA) sequences in these regions. Current results are similar to data that previously reported a high GC percentage in the IR regions, which could be due to the presence of ribosomal RNA (Qian et al., 2013).

**Table 1:** **Summary of complete chloroplast genomes for five *Nicotiana* species**.

	***N. otophora***	***N. sylvestris***	***N. tabacum***	***N. tomentosiformis***	***N. undulata***
Total	156,073	155,941	155,943	155,745	155,863
Large single copy (LSC, bp)	86,621	86,684	86,686	86,392	86,633
Inverted repeat (IR, bp)	25,888	25,342	25,343	25,429	25,331
Small single copy (SSC, bp)	17,677	18,573	18,571	18,495	18,568
GC%	37.7	37.8	37.8	37.8	37.9
Total	163	140	144	140	156
Protein coding genes	110	111	98	111	110
tRNA	45	37	37	37	37
rRNA	8	8	8	8	8

A total of 163 genes were found in the *N*. *otophora* cp genome, of which 116 are unique, including 110 protein-coding genes, 45 tRNA genes, and 8 rRNA genes (Figure 1, Table 1). Fourteen protein coding, four rRNA, and nine tRNA genes are repeated in the IR regions. The LSC region comprises 96 protein coding and 26 tRNA genes, whereas the SSC region comprises 15 protein-coding genes and 1 tRNA gene. The protein-coding genes present in the *N. otophora* cp genome include nine genes for large ribosomal proteins (*rpl2, 14, 16, 20, 22, 23, 32, 33, 36*), 11 genes for small ribosomal proteins (*rps2, 3, 7, 8, 11, 12, 14, 15, 16, 18, 19*), 5 genes for photosystem I (*psaA, B, C, I, J*), and 10 genes related to photosystem II. Furthermore, there are *six* genes (*atpA, B, E, F, H, I*) for ATP synthase and the electron transport chain in the *N*. *otophora* cp genome (Table 2). A similar pattern of protein coding genes was also shown by Sugiyama et al. (2005) and Yukawa et al. (2006) for *N. tabbacum* and *N. sylvestris*, respectively.

**Table 2:** **Genes in the sequenced *N. otophora* chloroplast genome**.

**Category**	**Group of genes**	**Name of genes**
Self-replication	Large subunit of ribosomal proteins	*rpl2, 14, 16, 20, 22, 23, 32, 33, 36*
	Small subunit of ribosomal proteins	*rps2, 3, 7, 8, 11, 12, 14, 15, 16, 18, 19*
	DNA dependent RNA polymerase	*rpoA, B, C1, C2*
	rRNA genes	*RNA*
	tRNA genes	*trnA-UGC, C-GCA, D-GUC, E-UUC, F-GAA, fM-CAU, G-UCC, H-GUG, I-CAU, L-CAA, M-CAU, N-GUU, P-GGG, P-UGG, Q-UUG, R-ACG, R-UCU, S-GCU, S-GGA, S-UGA, T-GGU, T-UGU, V-GAC, V-UAC, W-CCA, Y-GUA*
Photosynthesis	Photosystem I	*psaA, B, C, I, J*
	Photosystem II	*psbA, B, C, D, E, F, H, I, J, K*
	NadH oxidoreductase	*ndhA, B, C, D, E, F*
	Cytochrome b6/f complex	*petA, B, D, G, L, N*
	ATP synthase	*atpA, B, E, F, H, I*
	Rubisco	*rbcL, rbcLr*
Other genes	Translational initiation factor	*infA*
	Maturase	*matK*
	Protease	*clpP*
	Envelop membrane protein	*cemA*
	Subunit Acetyl- CoA-Carboxylate	*accD*
	c-type cytochrome synthesis gene	*ccsA*
Unknown	Conserved Open reading frames	*ycf1, 2, 3, 4, 15, 68*

**Figure 2:**
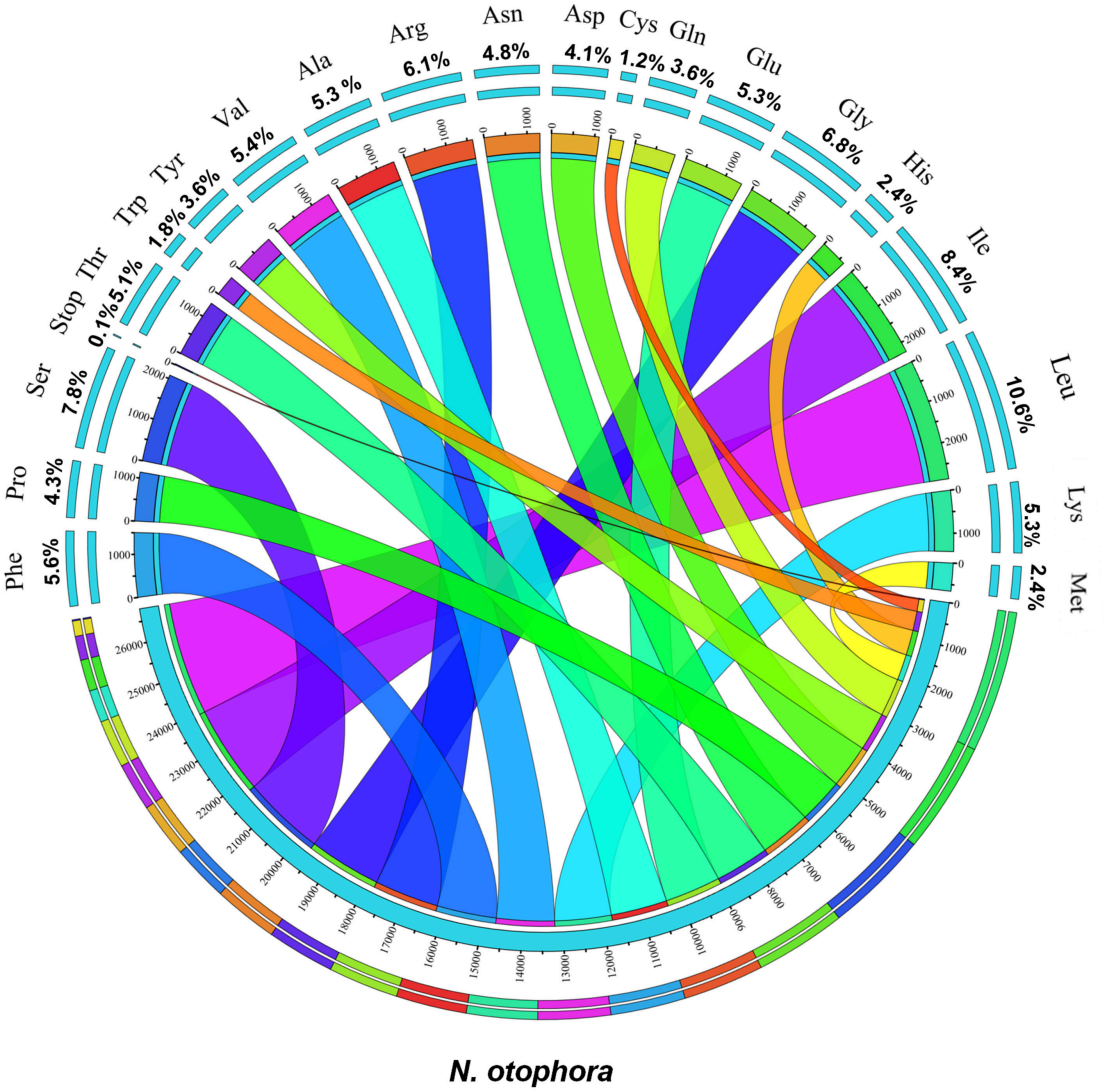
**Amino acid frequencies of the *N. otophora* cp protein coding sequences**. The frequencies of amino acids were calculated for all 110 protein-coding genes from the start to the stop codon.

Protein, rRNAs, and tRNAs are encoded by 51.5, 5.79, and 1.86% of the whole chloroplast genome, respectively, and the remaining 40.85% is non-coding regions. The 29 unique tRNA genes encode all of the 20 amino acids essential for protein biosynthesis. Furthermore, protein-coding sequences (CDS) are 80,379 bp in length and comprise 110 protein genes, which code for 26,793 codons (Tables 1, 3). The *N*. *otophora* cp genome codon usage frequency was determined by tRNA and protein-coding gene sequences (Table S1). Interestingly, leucine (10.6%) and cysteine (1.2%) were the maximum and minimum commonly coded amino acids, respectively (Figure 2). Among these, the maximum and minimum codons used were ATT (1087), encoding isoleucine, and ATT (1) encoding methionine, respectively. The AT content was 50.15, 61.72, and 66.83% at the 1st, 2nd, and 3rd codon positions within the CDS region (Table 3). The preference for a high AT content at the 3rd codon position is due to the A and T concentration reported in various terrestrial plant cp genomes (Morton, 1998; Tangphatsornruang et al., 2010; Nie et al., 2012; Qian et al., 2013).

**Table 3:** **Base composition in the *N. otophora* chloroplast genome**.

	**T/U**	**C**	**A**	**G**	**Length (bp)**
Genome	31.5	19.2	30.8	18.5	156,073
LSC	32.8	18.3	31.5	17.5	86,621
SSC	34.3	16.8	33.7	15.2	17,677
IR	28.6	22.3	28.4	20.7	25,888
tRNA	25.8	22.7	22.7	28.9	1268
rRNA	18.8	23.6	26.1	31.5	4524
Protein Coding genes	29.8	18.7	29.7	21.8	80,379
1st position	23.38	20.66	26.77	26.65	26,883
2nd position	32.38	20.34	29.4	17.89	26,883
3rd position	35.25	13.9	31.58	15.8	26,883

### Repeat analysis of *N. otophora* cp genome

**Figure 3:**
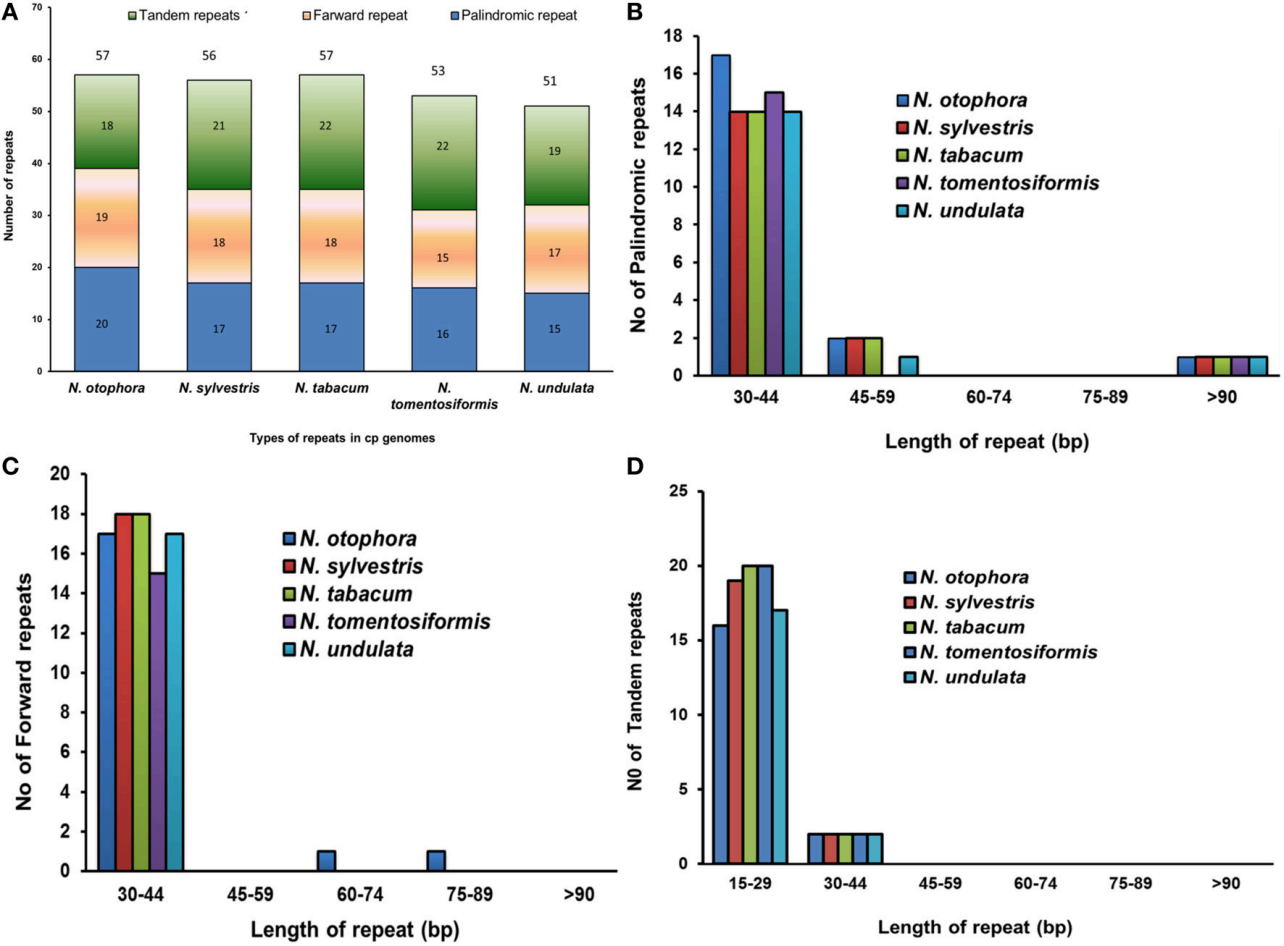
**Analysis of repeated sequences in five *Nicotiana* chloroplast genomes. (A)**, Total of three repeat types; **(B)**, Frequency of the palindromic repeat by length; **(C)**, frequency of the direct repeat by length; and **(D)**, Frequency of tandem repeat by length.

Repeat sequences are very helpful in phylogenetic study, and play a vital role in genome rearrangement (Cavalier-Smith, 2002; Nie et al., 2012). Furthermore, analysis of the various cp genomes concluded that repeat sequences are essential to induce indels and substitutions (Yi et al., 2013). For repeat analysis, 20 palindromic repeats, 19 forward repeats, and 18 tandem repeats were identified in the *N*. *otophora* cp genome (Figure 3A). Among these, 17 forward repeats had a size of 30–44 bp in length, whereas only two tandem repeats were found to be same length, and 16 were 15–29 bp in length (Figures 3A–D). Similarly, 17 palindromic repeats were 30–44 bp, and two repeats were 45–59 bp in length (Figure 3B). Overall, 57 repeats were found in the *N. otophora* cp genome. Similarly, 56, 57, 53, 51 repeat pairs were found in previously reported *N. sylvestris, N. tabacum, N. tomentosiformis*, and *N. undulata* (Figure 3A; Yukawa et al., 2006) genomes, respectively, when compared with *N. otophora* (Figure 3A). About 29.4% of these repeats were distributed in protein coding regions (Table S2). Previous reports suggested that sequence variation and genome rearrangement occurs due to the slipped strand mispairing and the improper recombination of these repeat sequences (Cavalier-Smith, 2002; Asano et al., 2004; Timme et al., 2007). Furthermore, the presence of these repeats indicates that the region is a crucial hotspot for genome reconfiguration (Gao et al., 2009). Additionally, these repeats are an informative source for developing genetic markers for phylogenetic and population genetics studies (Nie et al., 2012).

### SSR analysis of *N. otophora* cp genome

**Figure 4:**
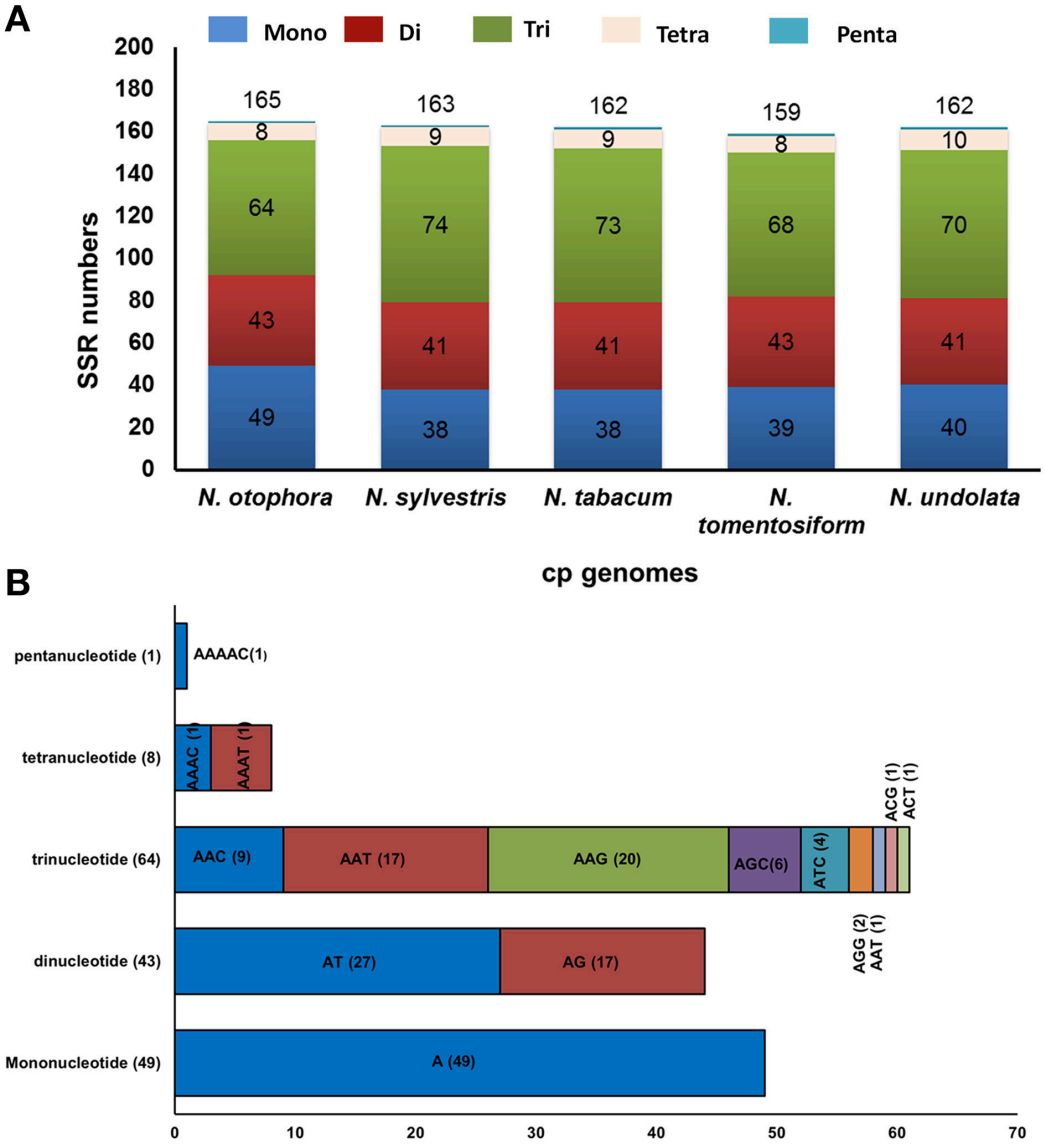
**Analysis of simple sequence repeat (SSR) in the five *Nicotiana* chloroplast genomes. (A)** Number different SSRs types detected in five genomes and **(B)** Frequency of identified SSR motifs in different repeat class types.

Simple sequence repeats (SSRs), or microsatellites, are 1–6 bp repeating sequences, which are distributed throughout the genome. Due to a high polymorphism rate at the species level, SSRs have been recognized as one of the main sources of molecular markers, and have been extensively researched in phylogenetic investigations and population genetics (Powell et al., 1995; Provan et al., 1997; Pauwels et al., 2012). In this study, we detected perfect SSRs over 10 bp in *N*. *otophora* together with four other *Nicotiana* species cp genomes (Figure 4A). Certain parameters were set, because SSRs of 10 bp or longer are prone to slipped strand mispairing, which is believed to be the main mutational mechanism for polymorphism (Rose and Falush, 1998; Raubeson et al., 2007; Huotari and Korpelainen, 2012). A total of 165 perfect microsatellites were analyzed in the *N. otophora* cp genome based on SSR analysis (Figure 4A). Similarly 163, 162, 159, and 162 SSRs were detected in *N. sylvestris, N. tabacum, N. tomentosiformis*, and *N. undulata*, respectively (Figure 4A). The majority of the SSRs in these cp genomes are mononucleotides, varying in quantity from 38 in *N. sylvestris* to 49 in *N. otophora*. Interestingly, trinucleotides are the second most predominant, ranging from 64 in *N. otophora* to 74 in *N. sylvestris*. Furthermore, only one pentanucleotide is present in all species (Figure 4A). In *N. otophora*, all mononucleotides (100%) are composed of A/T, and a similar majority of dinucleotides (61.36%) is comprised of A/T (Figure 4B). Our findings are comparable to previously reported arguments that SSRs found in the chloroplast genome are generally composed of polythymine (polyT) or polyadenine (polyA) repeats, and infrequently contain tandem cytosine (C) and guanine (G) repeats (Kuang et al., 2011). Therefore, these SSRs contribute to the AT richness of the *N. otophora* cp genome, as previously reported for different species (Kuang et al., 2011; Chen et al., 2015). SSRs were also detected in CDS regions of the *N*. *otophora* cp genome. The CDS account for approximately 51.50% of the total length. About 70.9% of SSRs are detected in non-coding regions, whereas only 26% of SSRs are present in the protein-coding region. Furthermore, about 2.42% of SSRs are present in the rRNAs and 0.6% was detected in tRNA genes. These results suggest an uneven distribution of SSRs in the *N. otophora* cp genome, which was also reported for different angiosperm cp genomes (Chen et al., 2015).

### Comparison of cp genomes of *N. otophora* and related *Nicotiana* species

**Figure 5:**
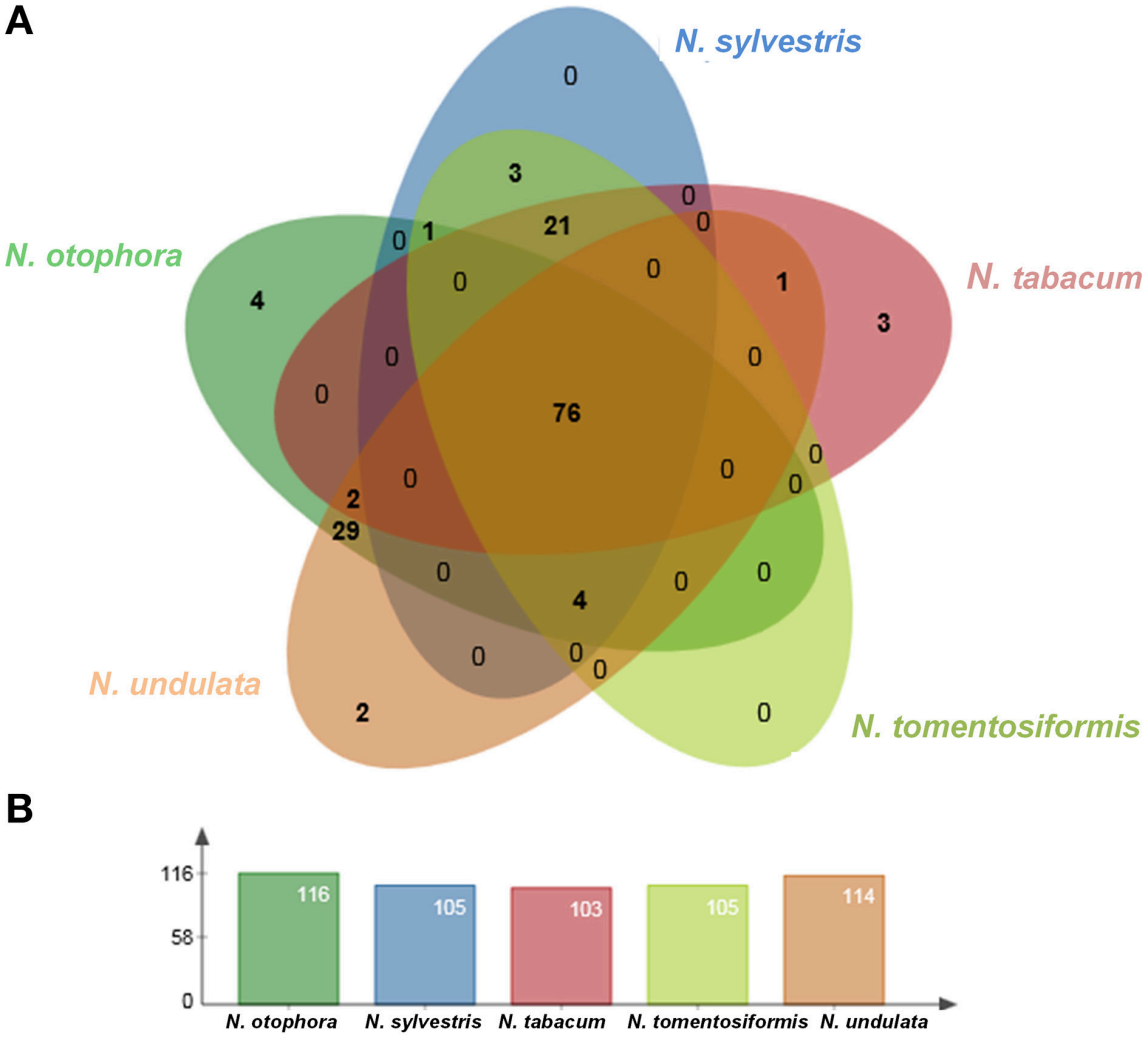
**Venn diagram illustrating the proportion of genes in five *Nicotiana* cp genomes. (A)** Number of protein coding genes shared by five *Nicotiana* cp genomes. **(B)** Number of unique genes identified in each cp genome.

Four complete cp genomes within the *Nicotiana* genus, namely *N*. *sylvestris* (155,941 bp), *N*. *tabacum* (155,943 bp), *N*. *tomentosiformis* (155,745 bp), and *N*. *undulata* (155,863 bp) were selected for comparison with *N*. *otophora* (156,073 bp). The genome size of *N*. *otophora* is the largest of these, and this difference is mostly attributed to the variation in the length of the IR region (Table 1). Analysis of genes with known functions showed that *N*. *otophora* shared 76 protein-coding genes, 20 tRNA genes, and 3 rRNA genes, with four other *Nicotiana* species cp genomes. The number of unique genes found in *N*. *otophora, N*. *sylvestris, N*. *tabacum, N*. *tomentosiformis*, and *N. undulata* cp genomes were 116, 105, 103, 105, and 114, respectively (Figure 5, Table S3).Furthermore, the overall gene organization and gene structures of these genomes were found very similar. However, some genes like, *cemA* and *infA* genes were found in *N. otophora, N. tabacum* and *N. undulata* while absent from *N. sylvestris and N. tomentosiformis* cp genomes. Similarly two genes *rbcLr* and *ycf68* were observed only in *N. otophora* genome (Table S4). The *ycf10* gene was absent in *N.otophora, N. tabacum* and *N. undulata* and founded in *N. sylvestris* and *N. tomentosiformis* cp genomes (Table S4).

**Figure 6:**
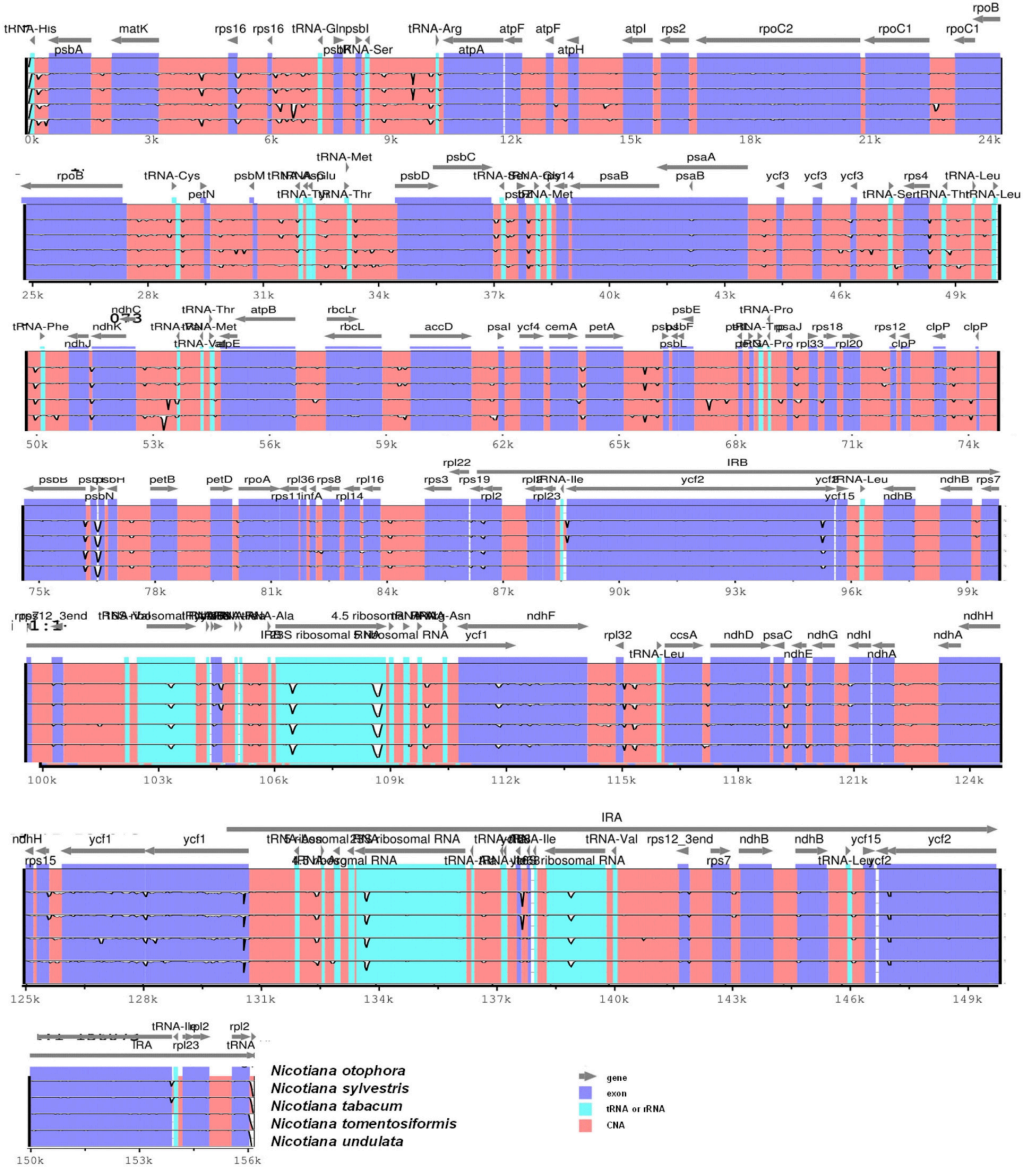
**Visualization alignment of five chloroplast genome sequences**. VISTA-based identity plot showing sequence identity among five *Nicotiana* species using *N. otophora* as a reference. The thick black line shows the inverted repeats (IRs) in the chloroplast genomes.

Pairwise cp genomic alignment between *N*. *otophora* with four other genomes uncovered a high degree of synteny. *N*. *otophora* annotation was used as a reference to plot the overall sequence identity of five *Nicotiana* species cp genomes using mVISTA (Figure 6). The results show that the LSC and SSC regions are more divergent than the two IR regions. Furthermore, non-coding regions exhibit a higher divergence than coding regions. These highly divergent regions include *ndhD, ndhH, ndhF, trnH-psbA, matK, ycF2, rpl22, rps15*, and *atpB* among others. Similar results related to these genes were reported previously (Qian et al., 2013), and the differences among various coding regions between species were also analyzed (Kumar et al., 2009).

### Genomes sequence divergence among *Nicotiana* species

**Figure 7:**
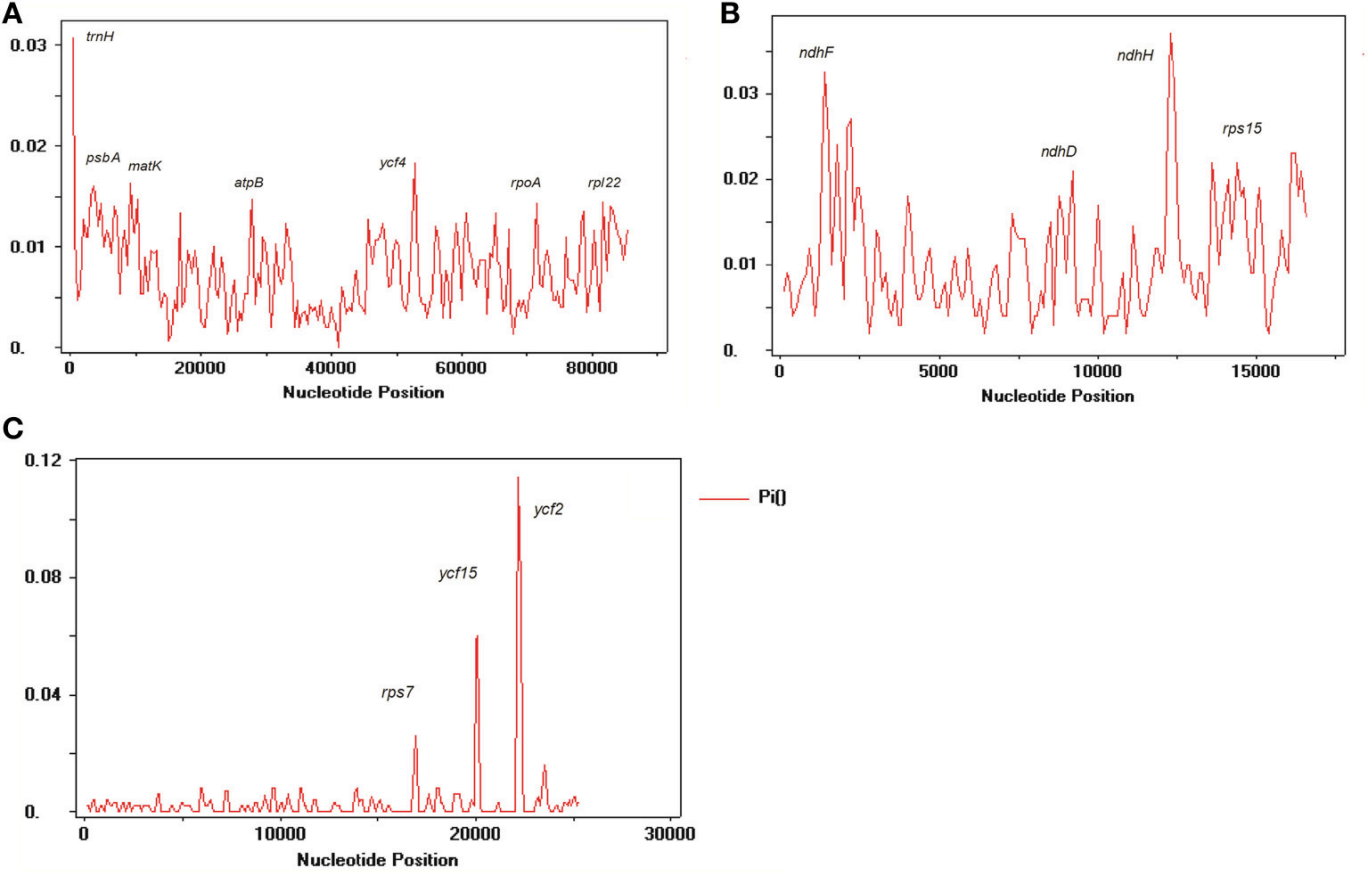
**Sliding window analysis of *N. otophora* with four *Nicotiana* cp genomes. (A)** Analysis of LSC regions, **(B)** Analysis of SSC regions, and **(C)** Analysis of IR regions. (window length: 600 bp, step size: 200 bp). X-axis, position of the midpoint of a window; Y-axis, nucleotide diversity of each window.

We compared the IR, LSC, and SSC regions in cp genomes and calculated the average pairwise sequence divergence among these five species. Of these regions, SSC had >0.010 average sequence divergence, and the most divergent region was found in *N. undulata* (0.0149). Among these three regions, IR has the least average sequence divergence (0.003) (Table S5). Furthermore, to calculate the sequence divergence level, the nucleotide variability (Pi) values within 600 bp in these five chloroplast genome LSC, SSC, and IR regions were calculated (Figure 7). In the IR region, these values varied from 0 to 0.1162 with a mean of 0.00216, the LSC region was from 0 to 0.030 with a mean of 0.0021, and the SSC regions were 0–0.1140, with a mean of 0.00321, indicating that the differences among these genome regions were small. However, some highly variable loci, including *trnA, psbA, matK, rps1, rps15, atpB, rpl22, rpl14, clpP, ndhF, ndhD, ndhH, ycF2, ycF4*, and *ycF15*, were more precisely located (Figure 7). All of these regions had much higher values than other regions (Pi > 0.007). Eight of these loci were located in the LSC region, four in the SSC region, and two were in the IR region. Among them, *psbA, clpP, matK, ndhF, rpl22, rps15, rpl14, ycF2*, and *ycF15* have been detected as highly variable regions in different plants (Kim and Lee, 2004; Dong et al., 2012; Qian et al., 2013). Based on these results, we believe that *ycf2, clpP, matK, rpl22, rps15*, and *ndhF*, which have comparatively high sequence deviation, are good sources for interspecies phylogenetic analysis, as previously reported (Chen et al., 2015).

### Phylogenetic analysis of *N. otophora* and related *Nicotiana* species cp genomes

**Figure 8:**
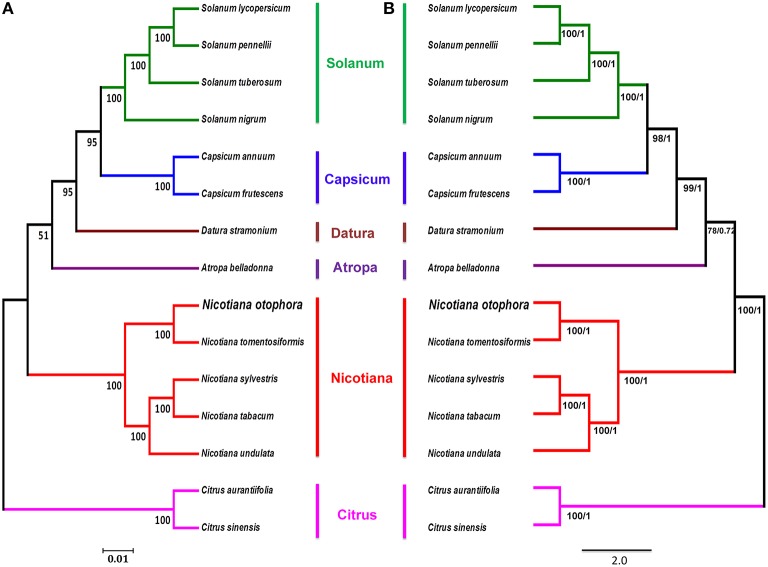
**Phylogenetic relationship of *N. otophora* with related species based on 75 protein-coding genes shared by all cp genomes**. Tree constructed by maximum likelihood **(A)**, maximum parsimony and Bayesian inference **(B)** with *Citrus aurantifolia* and *Citrus sinensis* as outgroups.

To study the phylogenetic position of *N. otophora* within the *Solanaceae* family, we used 75 protein-coding genes shared by the cp genomes of 13 *Solanaceae* members, representing five genera, for multiple alignments (Figure 8). Two species, *C. aurantifolia* and *C. sinensis*, were set as outgroups. Maximum likelihood (ML) analysis revealed 8 out of 11 nodes with bootstrap values ≥99%, and most of these nodes had 100% bootstrap values. For maximum parsimony (MP), the bootstrap values were very high for the MP tree, with values ≥99% for 10 of the 11 nodes. Both the ML and MP phylogenetic results were strongly supported, with 100% bootstrap values, and the position of *N. otophora* is clustered with *N. tomentosiformis* within *Nicotiana*, with *Atropha belladonna* and *Datura stramonium* as their closest relatives (Figure 8). Twelve species of *Solanaceae* from five different genera showed extremely conserved cp genome structures. In recent years, numerous studies employ cp DNA sequences to enrich phylogenetic analysis, which is substantially increasing our understanding of the evolutionary relationship between angiosperms (Leebens-Mack et al., 2005; Jansen et al., 2007; Moore et al., 2007).

## Conclusion

This study reported the complete chloroplast genome sequence of *N. otophora* (156,073 bp). The structure and organization of this genome is very similar to previously reported cp genomes from genus *Nicotiana*. The location and distribution of repeat sequences were detected, and LSC, SSC, and IR region sequence divergences were identified. Furthermore, MP and ML phylogenetic trees were constructed on the basis of protein coding genes, which were also shared by 12 *Solanaceae* members from five different genera. The data presented here will facilitate our understanding of the evolutionary history of tobacco. These findings provide a valuable analysis of the complete cp genome of *N*. *otophora*, which can be used to identify species, elucidate taxonomy, or reconstruct the phylogeny of the *Nicotiana* genus.

## Author contributions

All authors listed, have made substantial, direct and intellectual contribution to the work, and approved it for publication.

## Funding

This study was supported by a research fund (311058-05-3-CG000) from the Ministry for Food, Agriculture, Forestry, and Fisheries, Republic of Korea.

### Conflict of interest statement

The authors declare that the research was conducted in the absence of any commercial or financial relationships that could be construed as a potential conflict of interest.
