# Supplementary material for: Complete Chloroplast Genome of Nicotiana otophora and its Comparison with Related Species
Source: Front Plant Sci. 2016 Jun 14;7:843. doi: 10.3389/fpls.2016.00843 (PMC4906380; doi:10.3389/fpls.2016.00843)
Supplement: Supplementary file 1 [file DataSheet1.docx]

**TABLE S 1**| The codon-anticodon recognition pattern for the *N. otophora* chloroplast genome

| **Name** | **Type** | **Anticodon** | **Length** | **Direction** |  | **Name** | **Type** | **Anticodon** | **Length** | **Direction** |
| --- | --- | --- | --- | --- | --- | --- | --- | --- | --- | --- |
| *trnH* | His | GUG | 59 | Inverted |  | *trnT* | Thr | GGU | 59 | Direct |
| *trnI* | Ile | CAU | 74 | Direct |  | *trnM* | Met | CAU | 73 | Direct |
| *trnL* | Leu | CAA | 81 | Direct |  | *trnV* | Val | UAC | 38 | Inverted |
| *trnV* | Val | GAC | 72 | Inverted |  | *trnV* | Val | UAC | 37 | Inverted |
| *trnI* | Ile | GAU | 42 | Inverted |  | *trnF* | Phe | GAA | 73 | Direct |
| *trnI* | Ile | GAU | 35 | Inverted |  | *trnL* | Leu | UAA | 50 | Direct |
| *trnA* | Ala | UGC | 38 | Inverted |  | *trnL* | Leu | UAA | 37 | Direct |
| *trnA* | Ala | UGC | 35 | Inverted |  | *trnT* | Thr | UGU | 73 | Inverted |
| *trnR* | Arg | ACG | 74 | Inverted |  | *trnS* | Ser | GGA | 87 | Direct |
| *trnN* | Asn | GUU | 73 | Direct |  | *trnfM* | Met |  | 74 | Inverted |
| *trnL* | Leu | UAG | 80 | Direct |  | *trnG* | Gly | UCC | 71 | Direct |
| *trnN* | Asn | GUU | 73 | Inverted |  | *trnS* | Ser | UGA | 92 | Inverted |
| *trnR* | Arg | ACG | 74 | Direct |  | *trnM* | Met | CAU | 59 | Direct |
| *trnA* | Ala | UGC | 35 | Direct |  | *trnT* | Thr | GGU | 72 | Direct |
| *trnA* | Ala | UGC | 38 | Direct |  | *trnE* | Glu | UUC | 73 | Inverted |
| *trnI* | Ile | GAU | 35 | Direct |  | *trnY* | Tyr | GUA | 84 | Inverted |
| *trnI* | Ile | GAU | 42 | Direct |  | *trnD* | Asp | GUC | 74 | Inverted |
| *trnV* | Val | GAC | 72 | Direct |  | *trnC* | Cys | GCA | 72 | Direct |
| *trnL* | Leu | CAA | 81 | Inverted |  | *trnR* | Arg | UCU | 72 | Direct |
| *trnI* | Ile | CAU | 74 | Inverted |  | *trnS* | Ser | GCU | 88 | Inverted |
| *trnP* | Pro | GGG | 71 | Inverted |  | *trnQ* | Gln | UUG | 72 | Inverted |
| *trnP* | Pro | UGG | 74 | Inverted |  | *trnH* | His | GUG | 75 | Inverted |
| *trnW* | Trp | CCA | 74 | Inverted |  |  |  |  |  |  |
