# Supplementary material for: Complete Chloroplast Genome of Nicotiana otophora and its Comparison with Related Species
Source: Front Plant Sci. 2016 Jun 14;7:843. doi: 10.3389/fpls.2016.00843 (PMC4906380; doi:10.3389/fpls.2016.00843)
Supplement: Supplementary file 4 [file DataSheet4.docx]

**Table S4.** Occurrence of important genes in the cp genome of *N. otophora* in comparison with related *Nicotiana* species

| Genes | *N. otophora* | *N. sylvestris* | *N. tabacum* | *N. tomentosiformis* | *N. undulata* |
| --- | --- | --- | --- | --- | --- |
| *cemA* | *√* | *×* | *√* | *×* | *√* |
| *infA* | *√* | *×* | *√* | *×* | *√* |
| *rbcLr* | *√* | *×* | *×* | *×* | *×* |
| *ycf10* | *×* | *√* | *×* | *√* | *×* |
| *ycf68* | *√* | *×* | *×* | *×* | *×* |

*√ =* Genes common; × = gene not found
